# International Society for Extracellular Vesicles: first annual meeting, April 17–21, 2012: ISEV-2012

**DOI:** 10.3402/jev.v1i0.19995

**Published:** 2012-12-28

**Authors:** Elisa Araldi, Eva-Maria Krämer-Albers, Esther Nolte-‘t Hoen, Hector Peinado, Katarzyna Maria Psonka-Antonczyk, Pooja Rao, Guillaume van Niel, María Yáñez-Mó, Irina Nazarenko

**Affiliations:** 1 Department of Medicine and Cell Biology, Leon H. Charney Division of Cardiology, New York University School of Medicine, New York, USA; 2 Department of Molecular Cell Biology, Johannes Gutenberg University Mainz, Mainz, Germany; 3 Department of Biochemistry and Cell Biology, Utrecht University, Utrecht, The Netherlands; 4 Departments of Pediatrics, Cell and Developmental Biology, Weill Cornell Medical College, New York, USA; 5 Department of Physics, Norwegian University of Science and Technology, Trondheim, Norway; 6 Laboratory for Aging and Cognitive Diseases, European Neuroscience Institute, Göttingen, Germany; 7 Centre de Recherche, Institut Curie, UMR144 CNRS, Paris, France; 8 Hospital Santa Cristina, Instituto de Investigación Sanitaria Princesa, Madrid, Spain; 9 Department of Environmental Health Sciences, University Medical Centre, Freiburg, Germany

**Keywords:** extracelluar membrane vesicles, exosomes, microvesicles, microparticles, International Society for Extracellular Vesicles, ISEV-2012

## Abstract

Extracellular micro- and nano-scale membrane vesicles produced by different cells are recognised as an essential entity of physiological fluids in a variety of organisms and function as mediators of intercellular communication employed for the regulation of multiple systemic and local processes. In the last decade, an exponential amount of experimental work was dedicated to exploring the biogenesis and secretion mechanisms, physiological and pathological functions and potential applications of the extracellular vesicles (EVs). Noteworthy is the large heterogeneity of *in vitro* and *in vivo* models applied, technical approaches developed in these studies and the diversity of designations assigned to different or similar types of EVs. Hence, there is a clear necessity for a uniform nomenclature and standardisation of methods to isolate and characterise these vesicles. In April 2012, the first meeting of the International Society for Extracellular Vesicles (ISEV) took place bringing together this exponentially grown scientific community. The University of Gothenburg (Krefting Research Centre) together with the Interim Board of the Society created in September 2011 (Jan Lötvall, Clotilde Théry, Xandra Breakefield, Marca Wauben, Yong Song Gho, Lawrence Rajendran, Graça Raposo, Douglas Taylor, Margareta Sjöstrand and Esbjörn Telemo) organised this fantastic event that counted 488 registered and contributing participants. This meeting report provides a retrospective summary of the broad spectrum of ISEV-2012 sessions. Again, we emphasise novel findings, discussions and decisions met by the community during the meeting.

Intercellular communication by means of membrane-enclosed extracellular vesicles (EVs) attracted the attention of researchers in life sciences, clinics and pharmaceutical industry as an unexplored mechanism of molecule transfer, which can be applied as an ultimate tool in diagnostics and drug delivery. In April 2012, the first meeting of the International Society for Extracellular Vesicles (ISEV) took place fostering knowledge exchange within the large and diverse international community of this new exciting field. The main aims of the meeting were to highlight recent findings concerning the mechanisms of EV biogenesis and function, to present and to discuss techniques for the isolation of different types of EVs and for the separation of their content and to find a consensus for the nomenclature of different classes of EVs.

The conference was opened by *Olleco Larkö* (Dean Sahlegrenska Academy, Sweden); *Jan Lötvall* and *Clotilde Théry* (president and vice-president of the ISEV interim board, respectively) introduced the history of the ISEV establishment. The idea of a new community was born during the International Workshop on Exosomes (IWE), organised by Clotilde Théry and Graça Raposo in January 2011 in Paris. The name for the new community was defined on a democratic basis. Nowadays, the ISEV consolidate interests of researchers exploring different types of EVs, including exosomes, microvesicles, and other types of membrane vesicles released by cells into the extracellular environment.

*Douglas Mulhall* presented a historical retrospect of 50 years of EVs-related research, starting with the pioneering work of Dr. Anderson and Dr. Bonucci on the function of matrix vesicles in bone calcification, and extending to the role of vesicles in cancer etiopathology, rheumatoid and coronary diseases. Finally, Douglas Mulhall stressed the universal importance of the field for environmental sciences, fundamental understanding of various physiological and pathological processes in the organisms and for the creation and application of programmed and programmable artificial EVs as a natural drug delivery tool.

In this report, we adopted the vesicle nomenclature as used by the presenters. Furthermore, the designation “EVs” was included if different types of the extracellular membrane vesicles were discussed. Most articles cited in this report were either recently published, in press or published during the months following ISEV-2012. We apologise for not quoting all of the articles in this meeting report. All of the abstracts have been published as a supplement in the first issue of *Journal of Extracellular Vesicles* (JEV) and can be found on the JEV website: http://www.journalofextracellularvesicles.net/index.php/jev/article/view/18182/21587

## EVs as communication messengers: current state of knowledge and future perspectives

*Willem Stoorvogel* (Utrecht University, the Netherlands) summarised fundamental findings of the exosome research field, and specifically pointed out the importance of technical aspects, such as sucrose gradient ultracentrifugation, allowing for a reproducible separation of different vesicle subpopulations according to their size and buoyant density. Furthermore, he stressed the need to address in future the mechanisms of cargo incorporation into EVs. As an example, he presented data showing that the dendritic cells sort MHCII into exosomes in a complex with tetraspanins by a ubiquitin-independent pathway (1)

*Matthew Wood* (Oxford University, UK) emphasised advantages of the therapeutic application of exosomes for a specific delivery of RNA and other signalling molecules to different target organs. An elegant way to increase efficiency of delivery is to decorate exosomes with recombinant constructs consisting of an exosome-specific protein fused with a desired tissue receptor-specific ligand. For a therapeutic effect, exosomes can be electroporated with siRNA specific for certain disease-associated genes. Furthermore, Wood stressed a significantly higher capability of exosomes to pass the blood brain barrier as compared to currently used antibody-based drugs. The major challenge is to find an appropriate source of exosomes for clinical applications. As one possibility, Wood introduced a method currently used in animal trials, which is based on the isolation of haematopoietic progenitor cells, their genetic manipulation followed by *in vitro* exosome production, electroporation and intranasal or intravenous injection (2). As an alternative, stem cells and induced progenitor cells (iPCs) can be a valuable exosome source.

*Xandra Breakefield* (Massachusetts General Hospital, USA) discussed the application of EVs as a source for RNA cargo and its potential for the diagnosis and prognosis of cancer. Recent research demonstrated that in contrast to healthy individuals, plasma of cancer patients is enriched in exosomes and other types of EVs, containing mRNA, miRNA, ncRNA, rRNA, DNA and retrotransposon elements (3,4). The main challenge remaining is to define specific biomarkers, which can improve the accuracy of cancer diagnosis.

In summary, future perspectives and challenges in the biomedical applications of EVs are to complete a full characterisation of cargo of distinct populations of EVs derived from different sources. This should allow EV application for diagnosis and prognosis of various diseases. Furthermore, it is necessary to deepen knowledge and to develop new technological approaches for the use of EVs as natural specific and biocompatible vehicles for specific drug delivery and gene targeting *in vivo*.

## Mechanisms of EVs production

Regarding the biogenesis of EVs, the following aspects are critical for our fundamental understanding of EVs as carriers of signalling molecules: (a) the mechanisms of formation of different types of EVs; (b) the mechanisms behind sorting certain proteins, nucleic acid species and other molecules into EVs; (c) the regulation of the secretion/budding of EVs by the cells. It is also important to link the EV-related mechanisms with well-characterised cellular processes: internalisation, recycling, lysosomal degradation, cell division and fusion. Consensus in the community on the definition of EV subtypes and their production should be achieved to facilitate the implementation of this knowledge in understanding the role of EVs in physiological and pathological processes, for example, development, aging, inflammation, immunity, and cancer.

*Stephen Gould* (John Hopkins School of Medicine, USA) introduced the role of post-translational modifications and ESCRT machinery in the biogenesis of exosomes. Fundamentally, many plasma membrane anchors, such as myristoylation and palmitoylation might target oligomeric cytoplasmic proteins to the exosomes. In contrast, PI(3)P-binding domains mediate targeting to the endosomal compartment but not to the plasma membrane. The inhibition of vps2 and vps4 components of the ESCRT machinery exhibited different effects and in some cases, opposite effects on the recruitment of CD63, AcylTyA and CD81 to exosomes (5). According to the data presented by *Maria Yanez-Mo* (Hospital Santa Cristina, Spain), tetraspanin-enriched microdomains mediate sorting of tetraspanin-associated molecules in to the exosomes. Interference with the ceramide pathway by inhibiting neutral sphingomyelinase mediated inhibition of EV production in oligodendrocytes, as demonstrated by the group of M. Simons (6) and used by *Eva-Maria Kramer-Albers* (University Mainz, Germany) (7). One of the challenging questions that remain largely unexplored relates to the mechanism of RNA delivery into EVs. *Okay Saydam* (Medical University of Vienna, Austria) described a “zipcode”-like 25 bp sequence in the 3′-nontraslated region of mRNA, detected in EVs. It is likely that a miR-1289-binding site incorporated in this sequence plays an important role in the transfer of the mRNAs in to EVs. An increase of miR-1289 in the cells correlated with an enhanced recruitment of the mRNAs, containing a zipcode-like sequence in EVs. This study provides the first important indication towards understanding how mRNA might be targeted to EVs.

## Isolation, visualisation and separation of EV populations: improvement of available techniques and development of new approaches

Because of their small size, investigations into EVs face certain technical limitations. Thus, their visualisation is mostly restricted to electron microscopy; a separation of pure populations of EVs is barely possible and is difficult to control. There is an obvious demand for an improvement in available techniques and the development of new approaches. However, recently developed commercial products have not gained broad recognition within the community. Most of the presented studies relied on the basic protocols of differential centrifugation and sucrose gradient purification, both delivering reliable and reproducible results. Here, we would like to mention several recently developed techniques and present a comparative analysis of the different approaches.

Nanoparticle tracking analysis (NTA) based on light scattering allows characterisation of vesicle population in term of size and number (Nanosight Ltd, UK). Despite the relatively high costs of the equipment and efforts still required to establish protocols for the reliable measurements, this technology is tested in many laboratories worldwide. *Marca Wauben* (Utrecht University, the Netherlands) introduced high-resolution flow cytometry as a powerful tool for quantitative and qualitative analysis of subpopulations of vesicles down to 70 nm in size. With this technique, integrated information on the number, size and, assuming that specific surface markers are available, surface molecules of EV can be acquired (8). *Els van der Vlist* (Utrecht University, the Netherlands) showed that even within one fraction of a sucrose gradient, a heterogeneous population of T cell–derived EVs can be detected using the high-resolution flow cytometry-based method (9,10). *Richard Simpson* (La Trobe University, Australia) presented a direct comparison of 3 different methods for the isolation of exosomes from cell culture supernatants: differential centrifugation, OptiPrep density gradient centrifugation and EpCAM immunoaffinity capture. The latter was identified as the most efficient technique for the isolation of a distinct EV population, harbouring an antigen of interest (11).

Several techniques showing higher precision levels of EVs visualisation were discussed. *Agnes Kittel* (Semmelweis University, Hungary) provided evidences for the artefacts produced by the visualisation of EVs using TEM (transmission electron microscopy) and SEM (scanning electron microscopy) as compared to Cryo-EM (electron cryomicroscopy). Cryo-EM, based on the snap-freezing of a sample, was also presented by the groups of *Alain Brisson* (University of Bordeaux, France), *Andrew Hill* (The University of Melbourne, Australia) and *Willem Stoorvogel* (Utrecht University, the Netherlands), as a reliable technique to obtain native structural details, avoiding artefacts produced during fixation or drying. *Hjalmar Brismar* (Karolinska Institute, Sweden) discussed 3 types of super-resolution microscopy: stimulated emission depletion microscopy (STED), stochastic optical reconstruction microscopy/photoactivated localisation microscopy (STORM/PALM) and structured illumination microscopy (SIM) reaching resolution down to tens of nanometers. According to Brismar, super-resolution imaging is still limited by sample-related parameters such as the life-time of fluorochromes and the relatively large size of antibodies (15 nm). There is a need for the development of improved fluorescent staining reagents. As an alternative method, membrane-targeted recombinant fluorescently tagged proteins can be used for the visualisation of EVs, as presented by *Charles Lai* (Harvard Medical School, USA).

*Julie Hardij* (University of Namur, Belgium) introduced AFM (atomic force microscopy) as an alternative method for the visualisation of EVs. Using this technique, the visualisation of a single EV and of the specific surface antigens was possible. Raman microspectroscopy was presented by *Edwin van der Pol* (Amsterdam Academic Medical Center, the Netherlands) as an alternative for a label-free visualisation of EVs. Raman spectra of individual vesicles can be recorded. Prospectively, differentiation of EVs derived from various cellular origins (platelets, erythrocytes, tumour cells) should be possible. However, because of the high sensitivity of the method, standardisation of the sample preparation and establishment of standard spectra will be required. *Huilin Shao* (Harvard Medical School, USA) presented a magnetic nanoparticles-based real-time analysis of glioblastoma-derived exosomes. This method is a clever combination of microfluidic isolation of exosomes and magnetically labelled antibodies for the recognition of exosome markers by micro nuclear magnetic resonance. The method may be applied to monitor tumour progression or drug treatment efficiency. Further studies aiming at uncovering of EV-specific markers are necessary to evaluate possible future application of this technology in the clinic.

Nanotechnology offers alternative projections for the development of new approaches for EV purification and characterisation of their content. *Ryan Davies* (POSTECH, Korea) presented microfluidic cross-flow filtration as a method for the isolation of exosomes derived from the blood of melanoma-harbouring mice. *Kristan van der Vos* (Harvard Medical School, USA) introduced selective isolation of tumour microvesicles using a microfluidic chamber functionalised with antibodies. In this study, antibody-captured vesicles were lysed inside the chamber, allowing for a direct proceeding of the lysates for protein and RNA analysis. However, the capturing efficiency of the vesicles was reported to be only 10–20% thus needing improvement.

Specific and efficient isolation of distinct EVs components (RNAs, proteins and lipids) is one of the main technical challenges. *Maria Eldh* (University of Gothenburg, Sweden) reported a comparison of 7 different methods of the isolation of exosomal RNAs. Although no clear differences were observed in the purity of isolated RNA, the RNA yield and RNA size distribution were largely different when using different isolation techniques. Some methods yielded RNAs predominantly in the 20–200 nt range, whereas with other approaches 20–3,000 nt sized RNAs were isolated from the same source (12). These data raise awareness that a bias for specific RNA types may be introduced by the choice of the RNA isolation method.

To characterise the variety of nucleic acids in EVs, deep sequencing was applied by several research groups. *Danijela Koppers-Lalic* (Amsterdam University Medical Center, the Netherlands) described the results of small RNA deep sequencing in EBV-infected cells and corresponding exosomes and reported that virus infection leads to the loss or gain of specific miRNAs in the exosomes (13). *Esther Nolte-‘t Hoen* (Utrecht University, the Netherlands) presented the deep sequencing analysis of small RNA released during dendritic cell–T cell interactions. Notable findings were an abundance of several classes of non-coding RNA, such as tRNA fragments, UTR-derived small RNAs, small vault RNAs and a relatively small microRNA fraction (14). Additionally, to characterise microvesicle-specific mutations, *Leonora Balaj* (Massachusetts General Hospital, USA) described analysis of the glioma-derived microvesicle transcriptome by BEAMing qRT-PCR.

*Yiqi Seow* (Science and Engineering Institutes, Singapore) and *Igor V Kurochkin* (Bioinformatics Institute, Singapore) reported approaches for the packaging of nucleic acids into the exosomes. While packaging of plasmid DNA in exosomes is a challenge, the incorporation of aptamers by electroporation seems to be more efficient. Kurochkin described the results of a bioinformatic analysis of the RNA enriched in exosomes and specific motifs that could lead to the targeting of RNA to the exosomes. In the future, *in silico* modelling for the identification of EV-associated RNAs and their packaging might represent a highly attractive and challenging field for bioinformatics.

## EVs-mediated signal transfer in the neural network

Recent experimental data support a role of EVs in the physiological and pathological processes in the brain and their potential function as vehicles delivering signals from the periphery to the central nervous system (CNS). The majority of CNS cell types were shown to release exosomes and microvesicles. However, a direct link between cells producing EVs and the potential recipient cells internalising EVs has not been clearly demonstrated. Albeit many proteins involved in neurodegenerative processes were detected in the EVs, the functional role of EV secretion in the brain under homeostatic conditions as well as their potential mechanistic impact still remain open issues and were addressed by several groups during ISEV-2012.

*Kjell Fuxe* (Karolinska Institute, Sweden) presented a study suggesting that G-protein coupled receptors, for instance Adenosine A2A receptor, can be transmitted via microvesicles. Importantly, transmitted A2A receptor molecules were able to form heterodimers with other G-protein coupled receptors and function normally in the recipient cells. *Eva-Maria Krämer-Albers* (Johannes Gutenberg University Mainz, Germany) provided new evidence for the exosome-mediated neuron-glia communication in CNS. Her work indicated that neurons regulate exosome release from primary oligodendroglia by neurotransmitter signalling (7). The exosomes released by oligodendrocytes are subsequently internalised by neurons and microglia. In a sophisticated approach, the Cre-recombinase-based reporter system was utilised to demonstrate exosome transfer and functionality in the recipient cells. *Lawrence Rajendran* (University of Zurich, Switzerland) reported that the Amyloid *β*-peptide might be transported via exosomes. A high-resolution lipidomics study suggested an intracellular origin of Amyloid *β*-plaques and their inclusion into the exosomes. *Stefano Pluchino* (University of Cambridge, UK) reported that inflammatory cytokines affect miRNA and protein sorting into EVs derived from the neural precursor cells. Deep sequencing of the vesicular RNA revealed that these vesicles contain a selected set of miRNAs and that the miR* forms were depleted in the exosomes.

There is emerging evidence that exosomes cross the blood brain barrier under certain conditions and thus may be utilised as vehicles to deliver therapeutic agents to the brain. Matthew Wood's group demonstrated that dendritic cell–derived exosomes engineered to target neurons and equipped with therapeutic siRNAs are able to successfully silence genes in the brain after systemic injection (2). *Stefan Momma* (Frankfurt University Medical School, Germany) provided compelling *in vivo* evidence of exosome transfer from the hematopoietic cells over the blood brain barrier to CNS neurons using a Cre-LacZ-floxed mouse model. Selective targeting of Cre recombinase to peripheral hematopoietic cells resulted in an exosome-mediated transfer of the recombinase into the brain followed by the recombination of Purkinje neurons. The number of recombined neurons was increased after peripheral inflammation. *Huang-Ge Zhang* (University of Louisville, USA) presented that nasal administration of exosomes loaded with a reporter dye access the brain within minutes and are internalised by microglial cells. When the administered exosomes were loaded with an exogenous anti-inflammatory agent or a Stat3 inhibitor, mice were protected from neuroinflammation or exhibited reduced growth of a brain tumour, respectively (15). During the session, a discussion took place on whether EVs are present in the cerebrospinal fluid (CSF). There are apparent conflicting results on the quality and quantity of membrane particles in the CSF. *Roberto Furlan* (San Raffaele Scientific Institute, Italy) presented a comprehensive analysis of CSF microvesicles and demonstrated the presence of myeloid (microglia/macrophage-derived) microvesicles. Myeloid microvesicles were increased in CSF collected from multiple sclerosis (MS) patients during the active disease state and from a rodent model of acute MS. Such microvesicles appear to be actively involved in the disease process by spreading inflammatory signals. The study, which was performed in collaboration with *Claudia Verderio* (CNR Institute of Neuroscience, Italy), suggests that microvesicles in CSF may be valuable biomarkers for the diagnosis of MS and other neurodegenerative disorders.

## Role of EVs in pregnancy

Evidence for a role of EVs in physiological processes suggests that EVs might also contribute to the regulation of pregnancy. Consequently, alteration of the vesicles amount, size and content is likely to associate with abnormalities, such as preeclampsia, and could serve as biomarkers for diagnostic purposes. By using NTA and flow cytometry analysis, *Rebecca Dragovic* (University of Oxford, UK) showed general changes occurring in the profile of pregnant women compared to non-pregnant. EVs, especially platelet-derived microparticles, were increased in the blood of pregnant women compared to non-pregnant women. *Dionne Tannetta* (University of Oxford, UK) demonstrated that an increased size of microvesicles isolated from the placental perfusions correlates with preeclampsia. Furthermore, 5 miRNAs were detected and identified as specific preeclampsia markers.

## Role of EVs in tissue function, injury, repair and remodelling

Intercellular communication by mean of EVs seems to play an important role for the maintenance of tissue homeostasis. *Richard Simpson* (La Trobe University, Australia) addressed a potential contribution of EVs to the regulation of cell polarity by applying sequential immunocapture of exosomes released by the differentiated colorectal carcinoma cells using A33- and EpCAM-specific antibodies. Two different subpopulations of vesicles were identified: a basolateral (harbouring clathrin, AP1, AP3) type and an apical (positive for MUC13, Prom1) type, suggesting a connection between EVs release and cell polarisation.

Furthermore, it is likely that different types of EVs are involved in the regulation of tissue response to injury, for example, by protecting cells from apoptosis. *Paul Harrison* (Oxford Radcliffe Hospital, UK) reported a significant increase in the number of circulating microparticles (microvesicles released by platelets) after acute trauma and their impact on coagulopathy. *Claudia Bang* (Hannover Medical School, Germany) discussed the role of exosome miRNAs between cardiac fibroblasts and cardiomyocytes during cardiac remodelling, suggesting that fibroblast-derived miR-21* plays a role in cardiomyocyte hypertrophy. *Vince C De Hoog* (Utrecht University Medical Center, the Netherlands) showed that the protein levels of serum microvesicles associate with acute myocardial ischemia. Furthermore, he suggested potential novel biomarkers of ischemia. Moreover, a role for microvesicles was demonstrated for acute kidney injury. *Aldo Moggio* (University of Torino, Italy) reported that the miRNA in renal papillary CD133+ microvesicles released under hypoxic conditions can mediate renal recovery by protecting cells from apoptosis; *Stefania Bruno* (University of Torino, Italy) reported the role of mesenchymal stem cell–derived microvesicles in the induction of resistance to acute kidney injury via increased protection of renal tubular epithelial cells from apoptosis by an RNA-mediated mechanism.

EVs are likely to be involved in various pathological tissue remodelling processes, for example, tumour-related angiogenesis and vascular calcification. *Janusz Rak* (McGill University Health Centre, Canada) described how epithelial–mesenchymal transition (EMT) correlates with quantitative and qualitative changes in EVs. In a glioma model, he presented preliminary data, suggesting that EMT induces the production of tissue factor-carrying vesicles that are transferred to the endothelial cells, thereby inducing a pro-coagulant and metastasis-prone phenotype (16). *Yong Song Gho* (POSTECH, Korea) presented proteomic and genomic studies, showing a pro-angiogenic profile of colon cancer–derived EVs activating endothelial cells and macrophages by regulating the function of the early growth response protein 1 (EGR1). EGR1-specific siRNA and inhibition of MAPKinase were able to diminish the EV-mediated angiogenesis. *Jason Webber* (Cardiff University, UK) presented data supporting the transfer of TGF-β by exosomes derived from prostate cancer cells and their role in the induction of differentiation of fibroblasts into myofibroblasts, exhibiting a pro-angiogenic effect (17). These data further supported potential synergism of actions of growth factors and exosomes in the preparation of a pre-metastatic niche.

Vascular calcification seems to be regulated in an EV-dependent manner. Both vascular smooth-muscle cells (*Sundeep Kalra*, King's College London, UK) and macrophages (*Sophie New*, King's College, UK) released exosomes containing the inhibitor of calcification fetuin-A. Decrease of fetuin-A in the CD63 positive exosomes was associated with enhanced calcification. Additionally, *Alexander Kapustin* (King's College London, UK) showed that exosomes released from the vascular smooth-muscle cells regulate adenine nucleotide metabolism (18).

## Function of EVs in bone marrow

Hematopoietic cells release exosomes implicated in a variety of physiological and pathological processes, such as angiogenesis, coagulopathy and leukemic progression. *Susmita Sahoo* (Northwestern University, USA) presented *in vitro* and *in vivo* evidences for a pro-angiogenic role of miRNA-126 in exosomes derived from the CD34+ bone marrow (BM) progenitors (19). *Lewis R Goldberg* (Rhode Island Hospital, USA) presented data supporting that lung-derived EVs are capable of modulating the phenotype of BM progenitors by the regulation of cell cycle progression (20). This study pointed towards the role of cell cycle regulation defining the fate of BM progenitors.

*Etienne Moussay* (CRP-Santé, Luxemburg) and *Noah Hornick* (Oregon Health & Science University, USA) highlighted the importance of leukaemia-derived EVs (a cooperating action of exosomes and microvesicles or exosomes alone) in the regulation of the BM microenvironment in chronic lymphocytic leukaemia (CLL) and acute myeloid leukaemia (AML) models, respectively. Both studies supported the role of miRNA transfer via EVs regulating stromal BM cell behaviour. The LLC-derived exosomes and microvesicles seem to increase the survival and proliferation in stromal and endothelial cells by activation of MAPK, Akt, STAT3 and Src pathways. The AML-derived exosomes were enriched in certain miRNAs, which can be transferred between leukemic blasts and stromal cells. *Gerd Schmitz* (University Hospital Regensburg, Germany) presented a lipidomic characterisation of platelet-derived extracellular microvesicles and some of their potential physiological functions. His data indicated that the microvesicles accumulate precursors for eicosanoids and might be implicated in the regulation of inflammatory processes and cell proliferation. Additionally, his data suggest that the release of certain miRNA within the microvesicles contribute to platelet senescence.

## Role of EVs in immunity

The role of EVs in the regulation of immune response and inflammatory processes is widely recognised. Current studies are focused on further characterisation of EVs released by immune cells during physiological and pathological processes. Furthermore, many efforts are undertaken to understand the molecular mechanisms behind various functions of EVs in order to apply this knowledge in the development of EV-based therapies.

*Yi Lee* (University of Oxford, UK) described human induced pluripotent stem cell–derived dendritic cells (DCs) as a convenient source for EVs for therapeutic application. The vesicles were isolated from DCs at different differentiation stages (immature and mature DCs) and characterised with the help of NTA and western blot (flotilin-1 and CD9 as markers). The number of released exosomes was found to be dependent on the maturation state of DCs. *Jane Eastlake* (University of Bristol, UK) provided data suggesting that placental syncytiotrophoblast microparticles expressing foetal antigens are shed into maternal blood and could potentially be responsible for primary immune responses in neonatal alloimmune thrombocytopenia (NAIT). *Rebecca Martin* (Virginia Commonwealth University, USA) described how IgE immune complexes are transferred via CD23+ B cell–derived exosomes to DCs. The transferred antigens can be used by the recipient DCs for induction of T cell activation. *Tanja Näslund* (Karolinska Institute, Sweden) reported that DC-derived exosomes loaded with proteins are able to induce CD8+ T cells response in a B-cells dependent manner *in vivo*. *Salima Sadallah* (University Hospital Basel, Switzerland) reported that platelet-derived but not erythrocyte-derived ectosomes (plasma membrane–derived vesicles) induce proliferation of regulatory T cells (Tregs) via TGF-β transfer (21). *Lesley Ann Smyth* (MRC Centre for Transplantation, UK) showed that Tregs themselves produce exosomes with a suppressive activity. *Markus Peer* (University Hospital Regensburg, Germany) reported how in atherosclerosis, enzymatically modified low density lipoproteins (eLDL) induce the release of pro-apoptotic EVs from human neutrophils. These EVs were shown to induce apoptosis in macrophages. *Yang D. Dai* (Torrey Pines Institute for Molecular Studies, USA) showed that insulinoma cell lines secrete exosomes that induce a strong inflammatory response via induction of IL-6 and TNF-α and that such exosomes can contribute to insulitis (22). *Arun Cumpelik* (University Hospital Basel, Switzerland) in contrast, showed an immunosuppressive function of ectosomes derived from PMNs, based on their capacity to inhibit caspase-1 function. *Rabih El-Bizri* (Warren Alpert Medical School of Brown University, USA) described induction of hypertrophy and pulmonary vascular remodelling by the lung- and plasma-derived microparticles, suggesting their potential role in the regulation of monocrotaline-induced pulmonary hypertension.

## A “Trojan Horse” function of EVs during infection

In this session, the dualism of immune-regulatory and pro-inflammatory functions of exosomes became evident. *Caroline Gilbert* (University Laval, Canada) presented data supporting the immune-modulatory role of exosomes released by HIV-infected cells by showing their ability to induce apoptosis in the non-infected cells. *Philip Askenase* (Yale University School of Medicine, USA) showed that CD8+ T cells from tolerized mice released antigen-specific EVs containing miRNA-150 that suppressed IFNγ response. *Andrew Hill* (University of Melbourne, Australia) investigated exosomes in the relationship with prions. The abnormal prion isoforms PrPSc were found to be associated to exosomes released by the prion-infected cells. These data confirmed earlier findings showing that prion infectivity is associated with exosomes or other types of EVs (23,24). *Olivia Twu* (University of California, Los Angeles, USA) showed that *Trichomonas vaginalis* produces exosomes that contain parasite proteins affecting pathogenesis. By using a split-GFP assay, she demonstrated that exosome proteins from *Trichomonas* are successfully transferred to vaginal epithelial cells and may exhibit an immunosuppressive effect by diminishing the IL8-mediated inflammatory response of these cells to the parasite.

It is likely that exosomes or other types of EVs might act as “Trojan Horses” by mediating a direct transport of viral particles and prions or by contributing to the spread of parasite-derived proteins. Better understanding of the role of EVs during microbial infections and in the pathogenesis of infectious diseases may yield new diagnostic and therapeutic tools in the future.

## EVs in cancer

Evidence that EVs play a role in malignancies has been obtained in a variety of *in vitro* and *in vivo* models. Several ISEV-2012 sessions addressed different aspects of this issue and are summarised in this chapter.

*Kitty Agarwal* (Ohio State University, USA) presented lipidome analysis of exosomes isolated from the multiple myeloid leukaemia cell line U266, and their characterisation by asymmetric flow field flow fractionation. The preliminary analysis showed that the EVs are mainly below 200 nm and harbour different surface markers. *Ngai Na Co* (Anderson Medical Center, USA) demonstrated a relationship between the molecular content of exosomes and the resistance of ovarian cancer to chemotherapy. Adipocytes from ovarian cancer were analysed and miRNA-21 was shown to be horizontally transferred to ovarian cancer cells, resulting in enhanced taxol resistance and reduced apoptosis. Their data suggest that APAF1 could be the main target of miRNA-21 in this model. *Angelique Bobrie* (Institute Curie, France) presented the effects of knocking down Rab27a in the highly malignant cancer cell line 4T1. She demonstrated that inhibition of Rab27a reduced primary tumour growth and metastasis. This mechanism seems to be based on the reduction of exosome release and G-CSF secretion and the subsequent impairment of recruitment of neutrophils to primary tumours (25,26). *Alicia Llorente* (Oslo University Hospital, Norway) presented a preliminary characterisation of EVs from the prostate cancer cell line PC3 and described multiple proteins that potentially could be used in the prognosis of prostate cancer in the urine of patients. However, in the discussion the main idea was that the data available up to now are not sufficient to identify a good marker and further prostate cancer models, for example androgen receptor- dependent and -independent models need to be characterised (27). *Bow Tauro* (La Trobe University, Australia) introduced an impact of KRAS oncogene activation and of the EMT on the protein content of exosomes. The group conducted proteomics analysis on OptiPrep purified exosomes from epithelial MDCK cells and RAS-induced mesenchymal 21D1 cells to test whether exosomes could contribute to EMT. Tauro and colleagues observed an effect of the exosomes on extracellular matrix remodelling components, for example, proteases, splicing factors and migration factors. *Jeffrey L. Franklin* (Vanderbilt University Medical Center, USA) assessed the content of colon cancer-derived exosomes. He demonstrated that mutated KRAS can be horizontally transferred and promote the transformation of other cells. Additionally, amphiregulin can be transported via exosomes in a KRAS-dependent manner. Exosomal amphiregulin revealed higher activity than a recombinant protein (28). This study together with Al-Nedawi's work (29) demonstrates how the transfer of activated oncogenes by EVs could contribute to malignant cell transformation and cancer progression. *Andreas Baur* (University Clinic Erlangen, Germany) showed a model for TNF-α secretion by ADAM10 and TACE-loaded exosomes from HIV and melanoma patients. His model demonstrates that proteases such as ADAM10 or TACE process TNF-α inside MVB. In a combination of *in vitro* studies analysing the interaction network of NEF, ERK signalling, TACE/ADAM10 and TNF-α, he demonstrated the crucial role of exosomes in stimulating the release of the soluble factor TNF-α. *Hector Peinado* (Weil Cornell Medical School, USA) described the function of melanoma-derived exosomes in the metastatic niche priming. Experiments *in vivo* showed that tumour exosomes are mostly concentrated in those organs where metastases are formed: lung and BM cells. The latter contributed to metastases by a Met-dependent modulation of the BM progenitors (30).

## EVs as immune-modulators in cancer

Several reports suggested an immunomodulatory role of EVs in cancer progression. *Xin Tian* (National Center for Nanoscience and Technology, China) demonstrated that microvesicles can be used as a dual vaccine to prevent lung carcinoma and melanoma. Microvesicles derived from B16 melanoma cells and Lewis Lung Carcinoma (LLC) cells were used to treat immature DCs. Consequently, DCs were activated and exosomes were isolated from these cells and used for the immunisation of mice harbouring either LCC or B16 tumours. *Paul Robbins* (University of Pittsburgh School of Medicine, USA) showed that vaccination can also be accomplished with tumour-derived microvesicles and microvesicles isolated from the plasma in tumour bearing animals (31). *Jeroen de Vrij* (University Medical Center Utrecht, the Netherlands) analysed the effect of glioblastoma-derived exosomes on the behaviour and phenotype of polymorphonuclear cells (PMNCs) isolated from healthy donors. They showed CD14 up-regulation and HLA-DR down-regulation suggesting a shift to monocyte development and a blockade in dendritic and T cell differentiation. However, more studies are needed to verify this *in vitro* effect in glioblastoma patients. *Margot Zöller* (University Clinic Heidelberg, Germany) presented evidence that pancreas carcinoma exosomes exhibit multiple effects on leukocyte and lymphocyte behaviour. The uptake of tumour exosomes is mainly restricted to CD11b-positive cells and leads to possible impaired proliferation via reduced CD44v6 up-regulation, lck, ZAP70 and ERK-1,2 phosphorylation. *Peter J. Quesenberry* (Rhode Island Hospital, USA) showed a suppressive effect of prostate cancer–derived microvesicles on the cytotoxic effect of natural killer cells (NK). Microvesicles produced *in vitro* by the tumour cells differed in their activity depending on the cell passage. *Marie Lundholm* (Umea University, Sweden) demonstrated that tumour exosomes can down-regulate the expression of the receptor NKG2D in NK and CD8+ T cells, thereby inhibiting the NK phagocytic activity and inducing Fas-mediated apoptosis in lymphocytes. *Dhafer Mrizak* (Institute for Biology-Lille, France) reported a regulatory role of nasopharyngeal carcinoma–derived exosomes in the recruitment, expansion and activation of regulatory T cells. His study pointed towards the role of exosomes in intercellular transfer of cytokines. In the discussion, a question about a portion of cytokines distributed by exosomes as compared to the soluble fraction was addressed. *Ulf Gehrmann* (Karolinska Institute, Sweden) introduced an α-galactosylceramide dependent function of exosomes in activation of iNKT cells. Moreover, the administration of DC-exosomes containing α-galactosylceramide and ovalbumin in a model of OVA expressing melanoma resulted in a reduction of tumour growth and increased immune infiltration of the tumour. *Elisa Araldi* (New York University School of Medicine, USA) reported on the communication between macrophages and endothelial cells via exosomes. Vesicles were isolated from M1 macrophages and checked for specific markers (IL-16, IL-6, TNF). Upon stimulation with LPS, exosomes were enriched in microRNAs miR-155, miR-9, miR-147 and miR-16, the latter being highly enriched in the exosomes compared to the donor cells. Uptake of exosomes by endothelial cells resulted in a phenotype modification and down-regulation of VEGFR2 and FGFR1 expression.

## Application of EVs in diagnostics and treatment of malignancies

Due to the improvement of methods for the EVs isolation, characterisation and application of high-throughput techniques, different types of EVs derived from cancer cells or isolated from body fluids of cancer patients, were intensively characterised for their protein, lipid and RNA content. In this session, potential targets that could be used for diagnosis and prognosis of cancer progression were presented. Furthermore, new strategies for the manipulation of EV contents, properties and consequently functions were introduced and discussed.

*Wendy Westbroek* (National Human Genome Research Institute, Belgium) showed evidence that Rab27b promotes invasion and peritoneal metastasis of oestrogen receptor (ER) – positive breast cancer cells (32). The protein 8VATPase was identified to be closely linked to Rab27b vesicles. The combined presence of both Rab27b and 8VATPase are associated with poor prognosis in ER positive breast cancer. *Florian Dreyer* (University Clinic Erlangen, Germany) presented a preliminary characterisation of microRNA profiles isolated from the melanoma cells derived from patients. Their analysis showed that stage III and IV melanoma patients exhibit an increase of microRNAs packaged in circulating exosomes. Although they pointed out some specific miRNAs, more data will be required to identify a melanoma-specific profile. *Ulrich Putz* (The University of Melbourne, Australia) presented a study of exosome-mediated transfer of the tumour suppressor protein PTEN. Together with colleagues, he demonstrated that the NEDD4 family-interacting protein 1 (Ndfip1) regulates transfer of PTEN into the nucleus, promoting neural survival. Furthermore, it is required for the recruitment of PTEN into the exosomes. In patients with glioblastoma, the PTEN content in the exosomes was increased and correlated with Akt activation (33).

*Massimo Sargiacomo* (‘Charles Darwin’ La Sapienza University, Italy) and *Huilin Shao* (Massachusetts General Hospital, USA) characterised brain tumour–derived exosomes and presented a real-time monitoring system. Sargiacomo presented proteomic characterisation of neuroblastoma exosomes and preliminary evidence for transmissible acetylcholine signalling by these vesicles. Shao presented a magnetic nanoparticles-based real-time analysis of glioblastoma-derived exosomes. This method is a smart combination of microfluidic and micro nuclear magnetic resonance techniques, allowing for fast and specific vesicle detection. Further studies to find specific markers are necessary to ensure future application of this technology in the clinic. *Cecilia Lasser* (University of Gothenburg, Sweden) presented an initial study for the characterisation of exosomes from the isolated hepatic perfusion of uveal melanoma patients. Several exosome-associated miRNAs were identified as potential biomarkers.

## Biorepository

Analysis of EVs content by high-throughput approaches has allowed resulted in us gaining more information about lipids, proteins, mRNA and miRNA associated with EV. Next generation sequencing techniques allowed for the identification of new types of nucleic acids in EVs. It is becoming obvious that a platform allowing for storage, easy access and analysis of available data is required. Two different databases aiming at incorporating available information about the EV transcriptome, proteome and lipidome were presented during the ISEV meeting. *Yong Song Gho* (POSTECH, Korea) presented EVpedia allowing for an interspecies comparison of vesicular proteins starting from prokaryotic EVs to humans (http://evpedia.info). Exocarta, developed by *Suresh Mathivanan* in the group of *Richard Simpson* (La Trobe University, Australia), is well known in the community (34). Richard Simpson presented an extended version, called Vesiclepedia (http://exocarta.org/vesiclepedia). This database contains protein, lipid, miRNA and mRNA data of different types of EVs. Both Yong Song Gho and Richard Simpson asked for a collaborative involvement of research groups to include in the databases all the mandatory information about the EV isolation: source, number of passage by the cell lines and primary cells, details of the analyses procedures and so on. A community annotation of Vesiclepedia is currently in preparation.

## Nomenclature of different types of EVs

A common nomenclature of different types of EVs is one of the central issues discussed during ISEV-2012. In this session, *Graça Raposo* and *Stephen Gould* presented the current problems in EV nomenclature and discussed how to deal with this issue going forward. The ISEV community requires a consensus for a clear nomenclature of EVs. However, it seems to be far off from the consensus at this time. Many designations used for EVs, for example, oncosomes, ectosomes and so on, are based on either their biological effect or the cell type of origin. Moreover, clear criteria with regard to EV isolation and characterisation have not been defined up to now, and the lack of specific markers for the different types of vesicles is hindering this progress. *Jan Lötvall* pointed out the necessity of a consensus for the progress and recognition of the ISEV community in a broad scientific field. To allow reviewers and editors to adequately judge submitted manuscripts, clear criteria need to be identified with regard to nomenclature, markers and methods. One of the tasks identified by the community was the evaluation of the nomenclature issue, and the establishment of clear criteria allowing to find a consensus. Currently, the term “extracellular vesicles” is accepted as a general term to describe all types of vesicles released by different cell types under different conditions.

## NIH reaches out to ISEV

ISEV-2012 participants enjoyed an important opportunity to talk directly to NIH representatives *Elisabeth Wilder* and *her colleagues* and to discuss why research on EVs is a promising research field and about the possibilities for NIH to support its further development. From these discussions, it became clear that 4 main aspects of EVs should be addressed during the next 5 years to ensure progress of this research field: (a) understand the biogenesis of different types of EVs and develop methods allowing for their visualisation and discrimination; (b) determine the targets and functions of EVs; (c) characterise the content of human EVs in various body fluids; and (d) establish the clinical utility of EVs. The ISEV community thank the NIH representatives for the valuable and encouraging input and for their time dedicated to ISEV-2012. On 3 August 2012, NIH posted a new call “Clinical Utility of Extracellular RNA for Therapy Development”, developed as a NIH common fund initiative. We firmly believe that the EV research field needs the support of NIH to investigate and establish the application of EVs in human disease diagnosis, prognosis and prevention.

## General assembly

The general assembly of the community saw the official election of the new board of ISEV, which was proposed upon web-based voting by the ISEV members in February 2012. Subsequently, other society-related issues were discussed and future directions for the development of the community were defined. *Lawrence Rajendran* briefly presented the establishment of a Facebook community, which counted 308 members in April 2012. *Clotilde Théry* presented a summary and identified the following milestones achieved by the community: (a) setting up a Facebook page (Lawrence Rajendran); (b) several Survey Monkeys (Jan Lötvall) aimed at reaching consensus regarding the name and the logo for the community; and (c) launching of the JEV with Clotilde Théry, Peter Quesenberry, Yong Song Gho as co-Editors-in-Chief. The first issue of JEV, edited by Jan Lötvall, was published the day before ISEV-2012, and contained 6 original articles (9,25,34–37) in addition to all abstracts from the meeting. Furthermore, the ISEV-2013 meeting planned for 17–20 April in Boston was announced.

Several task forces were identified: (a) consensus for the nomenclature; (b) standardisation of protocols for EV isolation; (c) creation of EV databases and (d) RNA analysis. The latter task force is directed by Jan Lötvall and Marca Wauben, who organised a workshop on EV RNA isolation and analysis in New York in October 2012. Taskforces on EV-based biomarkers and cross-kingdom EVs may be established in the future.

## References

[1] Buschow SI, Nolte-‘t Hoen EN, van Niel G, Pols MS, ten Broeke T, Lauwen M (2009). MHC II in dendritic cells is targeted to lysosomes or T cell-induced exosomes via distinct multivesicular body pathways. Traffic.

[2] Alvarez-Erviti L, Seow Y, Yin H, Betts C, Lakhal S, Wood MJ (2011). Delivery of siRNA to the mouse brain by systemic injection of targeted exosomes. Nat Biotechnol.

[3] Noerholm M, Balaj L, Limperg T, Salehi A, Zhu LD, Hochberg FH (2012). RNA expression patterns in serum microvesicles from patients with glioblastoma multiforme and controls. BMC Cancer.

[4] Balaj L, Lessard R, Dai L, Cho YJ, Pomeroy SL, Breakefield XO (2011). Tumour microvesicles contain retrotransposon elements and amplified oncogene sequences. Nat Commun.

[5] Shen B, Wu N, Yang JM, Gould SJ (2011). Protein targeting to exosomes/microvesicles by plasma membrane anchors. J Biol Chem.

[6] Trajkovic K, Hsu C, Chiantia S, Rajendran L, Wenzel D, Wieland F (2008). Ceramide triggers budding of exosome vesicles into multivesicular endosomes. Science.

[7] Fruhbeis C, Frohlich D, Kramer-Albers EM (2012). Emerging roles of exosomes in neuron-glia communication. Front Physiol.

[8] Hoen EN, van der Vlist EJ, Aalberts M, Mertens HC, Bosch BJ, Bartelink W (2012). Quantitative and qualitative flow cytometric analysis of nanosized cell-derived membrane vesicles. Nanomedicine.

[9] van der Vlist EJ, Arkesteijn GJA, van de Lest CHA, Stoorvogel W, Nolte-‘t Hoen ENM, Wauben MHM (2012). CD4+ T cell activation promotes the differential release of distinct populations of nanosized vesicles. J Extracellular Vesicles.

[10] van der Vlist EJ, Nolte-‘t Hoen EN, Stoorvogel W, Arkesteijn GJ, Wauben MH (2012). Fluorescent labeling of nano-sized vesicles released by cells and subsequent quantitative and qualitative analysis by high-resolution flow cytometry. Nat Protoc.

[11] Tauro BJ, Greening DW, Mathias RA, Ji H, Mathivanan S, Scott AM (2012). Comparison of ultracentrifugation, density gradient separation, and immunoaffinity capture methods for isolating human colon cancer cell line LIM1863-derived exosomes. Methods.

[12] Eldh M, Lötvall J, Malmhäll C, Ekström K (2012). Importance of RNA isolation methods for analysis of exosomal RNA: evaluation of different methods. Mol Immunol.

[13] Koppers-Lalic D, Hogenboom MM, Middeldorp JM, Pegtel DM (2012). Virus-modified exosomes for targeted RNA delivery; a new approach in nanomedicine. Adv Drug Deliv Rev.

[14] Nolte-‘t Hoen EN, Buermans HP, Waasdorp M, Stoorvogel W, Wauben MH, ‘t Hoen PA (2012). Deep sequencing of RNA from immune cell-derived vesicles uncovers the selective incorporation of small non-coding RNA biotypes with potential regulatory functions. Nucleic Acids Res.

[15] Zhuang X, Xiang X, Grizzle W, Sun D, Zhang S, Axtell RC (2011). Treatment of brain inflammatory diseases by delivering exosome encapsulated anti-inflammatory drugs from the nasal region to the brain. Mol Ther.

[16] Garnier D, Magnus N, D'Asti E, Hashemi M, Meehan B, Milsom C (2012). Genetic pathways linking hemostasis and cancer. Thromb Res.

[17] Webber J, Steadman R, Mason MD, Tabi Z, Clayton A (2010). Cancer exosomes trigger fibroblast to myofibroblast differentiation. Cancer Res.

[18] Kapustin AN, Shanahan CM (2012). Calcium regulation of vascular smooth muscle cell-derived matrix vesicles. Trends Cardiovasc Med.

[19] Sahoo S, Klychko E, Thorne T, Misener S, Schultz KM, Millay M (2011). Exosomes from human CD34(+) stem cells mediate their proangiogenic paracrine activity. Circ Res.

[20] Aliotta JM, Lee D, Puente N, Faradyan S, Sears EH, Amaral A (2012). Progenitor/stem cell fate determination: interactive dynamics of cell cycle and microvesicles. Stem Cells Dev.

[21] Eken C, Sadallah S, Martin PJ, Treves S, Schifferli JA (2012). Ectosomes of polymorphonuclear neutrophils activate multiple signaling pathways in macrophages. Immunobiology.

[22] Sheng H, Hassanali S, Nugent C, Wen L, Hamilton-Williams E, Dias P (2011). Insulinoma-released exosomes or microparticles are immunostimulatory and can activate autoreactive T cells spontaneously developed in nonobese diabetic mice. J Immunol.

[23] Bellingham SA, Coleman BM, Hill AF (2012). Small RNA deep sequencing reveals a distinct miRNA signature released in exosomes from prion-infected neuronal cells. Nucleic Acids Res.

[24] Coleman BM, Hanssen E, Lawson VA, Hill AF (2012). Prion-infected cells regulate the release of exosomes with distinct ultrastructural features. FASEB J.

[25] Bobrie A, Colombo M, Krumeich S, Raposo G, Théry C (2012). Diverse subpopulations of vesicles secreted by different intracellular mechanisms are present in exosome preparations obtained by differential ultracentrifugation. J Extracellular Vesicles.

[26] Bobrie A, Krumeich S, Reyal F, Recchi C, Moita LF, Seabra MC (2012). Rab27a supports exosome-dependent and -independent mechanisms that modify the tumor microenvironment and can promote tumor progression. Cancer Res.

[27] Hessvik NP, Phuyal S, Brech A, Sandvig K, Llorente A (2012). Profiling of microRNAs in exosomes released from PC-3 prostate cancer cells. Biochim Biophys Acta.

[28] Higginbotham JN, Demory BM, Gephart JD, Franklin JL, Bogatcheva G, Kremers GJ (2011). Amphiregulin exosomes increase cancer cell invasion. Curr Biol.

[29] Al-Nedawi K, Meehan B, Micallef J, Lhotak V, May L, Guha A (2008). Intercellular transfer of the oncogenic receptor EGFRvIII by microvesicles derived from tumour cells. Nat Cell Biol.

[30] Peinado H, Alečković M, Lavotshkin S, Matei I, Costa-Silva B, Moreno-Bueno G (2012). Melanoma exosomes educate bone marrow progenitor cells toward a pro-metastatic phenotype through MET. Nat Med.

[31] Yang C, Ruffner MA, Kim SH, Robbins PD (2012). Plasma-derived MHC class II+ exosomes from tumor-bearing mice suppress tumor antigen-specific immune responses. Eur J Immunol.

[32] Hendrix A, Maynard D, Pauwels P, Braems G, Denys H, Van den Broecke R (2010). Effect of the secretory small GTPase Rab27B on breast cancer growth, invasion, and metastasis. J Natl Cancer Inst.

[33] Putz U, Howitt J, Doan A, Goh CP, Low LH, Silke J (2012). The tumor suppressor PTEN is exported in exosomes and has phosphatase activity in recipient cells. Sci Signal.

[34] Simpson RJ, Kalra H, Mathivanan S (2012). ExoCarta as a resource for exosomal research. J Extracellular Vesicles.

[35] Aliotta JM, Pereira M, Li M, Amaral A, Sorokina A, Dooner MS (2012). Stable cell fate changes in marrow cells induced by lung-derived microvesicles. J Extracellular Vesicles.

[36] de Jong OG, Verhaar MC, Chen Y, Vader P, Gremmels H, Posthuma G (2012). Cellular stress conditions are reflected in the protein and RNA content of endothelial cell-derived exosomes. J Extracellular Vesicles.

[37] Ekström K, Valadi H, Sjöstrand M, Malmhäll C, Bossios A, Eldh M (2012). Characterization of mRNA and microRNA in human mast cell-derived exosomes and their transfer to other mast cells and blood CD34 progenitor cells. J Extracellular Vesicles.

